# Development of a standardized testing system for orthodontic sliding mechanics

**DOI:** 10.1186/s40510-015-0087-8

**Published:** 2015-06-04

**Authors:** Maryam Fathimani, Garrett W Melenka, Dan L Romanyk, Roger W Toogood, Giseon Heo, Jason P Carey, Paul W Major

**Affiliations:** Orthodontic Department, School of Dentistry, University of Alberta, Edmonton, AB Canada; Department of Mechanical Engineering, University of Alberta, Edmonton, AB Canada; School of Dentistry, University of Alberta, Edmonton, AB Canada; Faculty of Medicine and Dentistry, 5-478, Edmonton Clinic Health Academy (ECHA), University of Alberta, 11405-87 Avenue NW, Edmonton, AB T6G 1C9 Canada

**Keywords:** Resistance to sliding, Friction, Orthodontics, Archwire/bracket interaction

## Abstract

**Background:**

The primary objective of this study was to develop a computer-controlled three-dimensional friction measuring system, the orthodontic friction simulator (OFS). A clinically-based in vitro experiment considering wet and dry friction for conventionally and self-ligated brackets is presented to elucidate debate surrounding sliding mechanics and illustrate capabilities of the OFS.

**Methods:**

The OFS was designed and manufactured using sound engineering principles and with the primary concern of being able to measure all forces and moments generated during sliding mechanics. This required the implementation of a six-axis load cell. A variety of translation and rotation stages were also incorporated to allow for precise positioning of the bracket relative to the archwire. Once designed and built, the OFS was then used to compare conventional and self-ligation methods in both the wet and dry state. Damon Q brackets and 0.018″ × 0.025″ stainless steel wires were used for all tests with a sample size of *n* = 65 for each ligation method. Archwires were pulled at a speed of 0.1 mm/s in 11 increments of 0.1 mm. At each increment, the bracket would be rotated 0.5° resulting in a total archwire travel of 1.1 mm and a second-order bracket angle range of 0°–5°. A repeated measures ANOVA was conducted to determine if ligation method and/or addition of moisture effected resulting orthodontic loads.

**Results:**

The developed equipment for studying orthodontic sliding mechanics was able to measure forces and moments in all three directions; a capability not previously realized in the literature. Additionally, it was found that passive ligation significantly reduced resistance to sliding, *P* ≤ 0.05, while the dry/wet state did not.

**Conclusions:**

The OFS certainly proved to be an adequate instrument for the scientific evaluation of orthodontic sliding mechanics. It is capable of measuring loads generated in all directions and is a fully automated apparatus allowing for simple and repeatable friction tests to be conducted. Furthermore, the addition of saliva was not found to significantly influence the loads generated during sliding mechanics regardless of ligation method.

## Background

Resistance to sliding (RS), or friction, in bracket-archwire interaction is of clinical interest in sliding mechanics. In the past, many studies were conducted on this subject [1–5]. Although this has been partly in response to marketing claims of new orthodontic appliances, interest in friction was present [1] long before these appliances were available. Despite this interest, there have been no proposed or adopted standards to measure or evaluate RS, in the oral or laboratory environment. This has contributed to a spirited discussion [6, 7] about the fundamental nature of friction and, separately, its role in the clinical environment.

The complicated frictional interaction, hence the friction coefficient, between two sliding surfaces cannot be deduced theoretically from first principles of physics. A numerical model [2] showed good agreement with experimental phenomena including time dependence of static friction, stick–slip, velocity weakening, and others that are relevant in orthodontic biomechanics. This model, however, must be adapted in an ad hoc manner to produce satisfactory results as experimental parameters are not known beforehand. For the present, knowledge of the friction interaction in any specific situation must be obtained by physical experiment.

An ongoing and common misconception [3] is that the force of friction is a constant fraction of the normal force. This is usually expressed as *F =* μ*N*, where *F* is the friction force, *N* is the normal force, and μ is an experimentally determined friction coefficient. This is true only under conditions of either impending or relative motion. For impending motion when the surfaces are on the verge of sliding, μ *=* μ_*s*_ is the coefficient of static friction, and *F = F*_max_ is the static friction limit. During relative motion when surfaces slide relative to each other, μ *=* μ_*k*_ is the coefficient of kinetic friction, and typically μ_*k*_ *<* μ_*s*_. Throughout the majority of treatment, however, there is minimal relative motion of the archwire and bracket. The friction, including its direction, is determined by conditions of equilibrium of all the forces acting on the tooth-bracket-archwire complex and will be less than the limiting value (e.g*.* 0 *≤ F ≤* μ_*s*_ *N*).

Clinically, the complete set of forces acting on the tooth is not known, therefore friction is also unknown. However, it is experimentally possible to determine the limiting value of friction since it is a property of the two surfaces involved. This is presumably the value that must be exceeded to allow relative motion between the archwire and bracket. The static limit is difficult to determine and recourse is made to measure μ_*k*_, with the understanding that it represents a lower bound on the static limit.

Nanda [4] listed over 20 variables and factors that affect this interaction in the mouth. Because of this complexity, measures of friction in the oral environment are very difficult and therefore extremely rare. Recourse has been made to in vitro experiments to examine key interactions. Previous bench-top studies have explored the effects of wire and bracket geometry [5, 8], material properties [9, 10], ligation method [11–15], tooth angulation [16–19], position of adjacent teeth [20, 21], effect of saliva [22–25], perturbation [26–28], and others. RS is the combination of several different effects, which become dominant at different angles of second-order rotation.

**Fig. 1:**
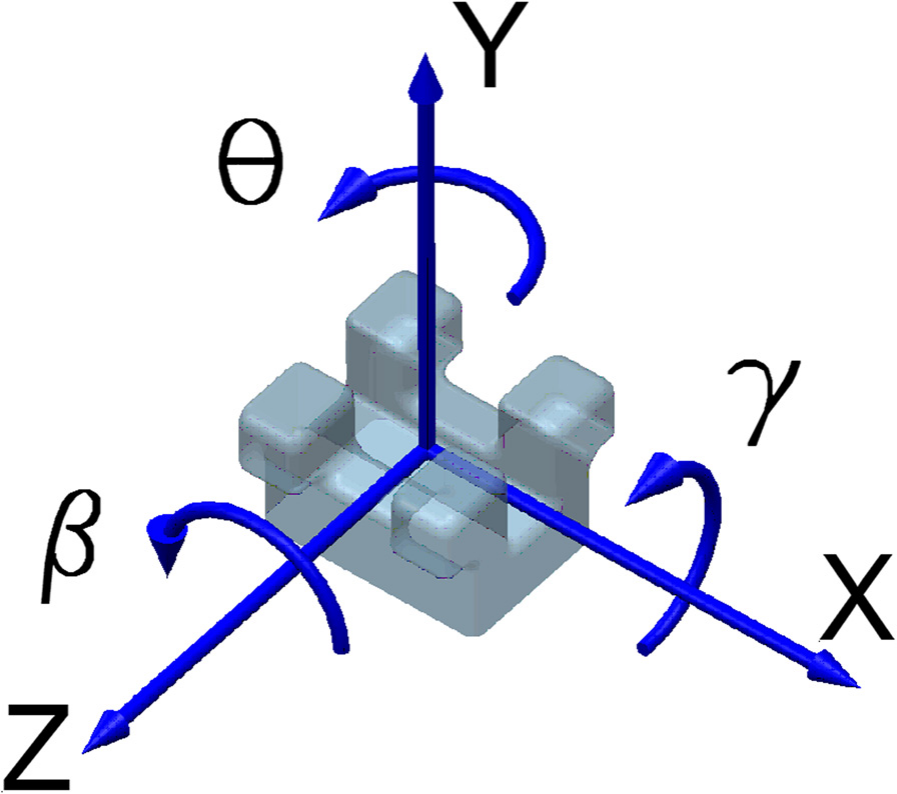
The bracket coordinate system CSYS and positive direction of forces, moments, and displacements

For the coordinate system (CSYS) attached to the bracket in Fig. 1, tip, or second-order angulation, is the angle θ. Kusy and Whitley [5, 9] suggested that RS is a combination of simple (classical) friction (FR), binding (BI), and/or notching (NO), expressed as, Eq. (1):

1 $$ \mathrm{R}\mathrm{S}=\mathrm{F}\mathrm{R}+\mathrm{BI}+\mathrm{NO} $$

*FR* occurs at low tip angles due to the force pressing the wire onto one of the sides or back of the slot wall or due to the gripping of the wire by the ligation. For low angulation, *FR* is only mildly affected by the tip angle.

When the tip angle exceeds some critical value θ_*c*_ [5], BI is the dominant interaction. The wire contacts opposite (medial-distal) edges at each end of the slot such that there are opposing contact forces in the ±Z direction. The two opposing normal forces produce a couple *M*_*y*_. If the opposing forces are equal, there is no net force in the Z direction. Non-equal opposing forces will yield a net force and friction, thus *FR* is still present. The large opposing forces on the corners of the bracket will cause a frictional resistance parallel to the slot even if the net force is zero. The friction at these contact points is therefore related to the magnitude of the couple; if the wire is linearly elastic (for example, stainless steel (SS)), the friction increase is proportional to the tip angle of the bracket beyond θ_*c*_. For a given couple *M*_*y*_, the friction force will increase for narrower brackets [29]. That is, if *M*_*y*_ remains constant but the bracket width decreases, then the normal force must increase. As friction is proportional to the normal force, it follows that friction will also increase in this scenario.

At sufficiently high angles of tip, physical interlocking of the wire and slot edges caused by plastic (i.e., permanent) deformation of the wire or bracket and/or other surface damage will cause very high, non-friction-based resistance; this is called notching (*NO*). Above the critical notching angle θ_*z*_, *RS* increases unpredictably and very quickly to the extent that sliding likely ceases. For this reason, the *NO* phenomenon is not of great clinical interest.

The vast majority of laboratory experiments investigating orthodontic friction only record one-dimensional (1D) frictional data (i.e., the force required to pull the wire; nominally *F*_*x*_). As such, the component breakdown of forces at the bracket is not understood, nor are any couples generated during sliding. The apparatus of Kusy and Whitley [5] measured the applied normal force perpendicular to the back wall of the slot at the same time as *F*_*x*_. The extreme variability of experimental methods used in the literature makes it difficult to compare results between friction studies. With no standardized test equipment or methods, often the results can be used only to draw qualitative conclusions within the limitations of the tests conducted (e.g., wire/bracket combination A versus combination B).

It was proposed by Kamelchuk and Rossouw [30] that an apparatus was required to obtain a more complete picture of orthodontic friction and “would allow a high standard of basic hypothesis testing, product development, quality control, and product performance evaluation with relative ease. (Kamelchuk and Rossouw [30])”. A series of design specifications included simultaneous control of velocity and acceleration of both linear wire position and angular bracket position and high-speed digital data acquisition. However, their prototype device [30] was capable only of measuring *F*_*x*_, as a uniaxial testing machine was used. The device did allow tensioning of the wire which affects its stiffness and therefore, ultimately, *RS*. By testing only a single bracket without lateral constraints on the wire caused by adjacent brackets, the important effect of interbracket distance was not included.

No apparatus measuring three-dimensional (3D) forces and moments on the bracket during friction tests has been published. Devices do exist that measure the 3D loads during other orthodontic treatment protocols; however, this is the first exclusively focusing on sliding mechanics. An understanding of loads in 3D space is essential [31] to effectively analyze tooth movements. There is certainly a relation between the normal forces and moments (caused either by ligation or wire stiffness) and RS; however, these other force and moment components have not been reported.

This paper describes the first published apparatus for measuring the complete 3D six components of force and moment (i.e., *F*_*x*_, *F*_*y*_, *F*_*z*_, *M*_*x*_, *M*_*y*_, and *M*_*z*_) system applied to the bracket during simulated retraction of a canine tooth in an extraction case. The apparatus supports the study of the following variables: bracket and wire geometry, materials, ligation method, dry/wet tests, direct force between wire and slot surface, and second-order angulation, or tip, of the tooth. With minor modifications, the apparatus can also support study of third-order rotation, or torque, buccal/lingual and coronal/apical misalignment, and interbracket distance. Since it is small and portable, the apparatus can be placed within an environment chamber to assess effects of temperature. The apparatus will be demonstrated here by exploring the effects of second-order angulation on forces and moments on conventional and self-ligating brackets in the dry and wet states.

## Methods

**Fig. 2:**
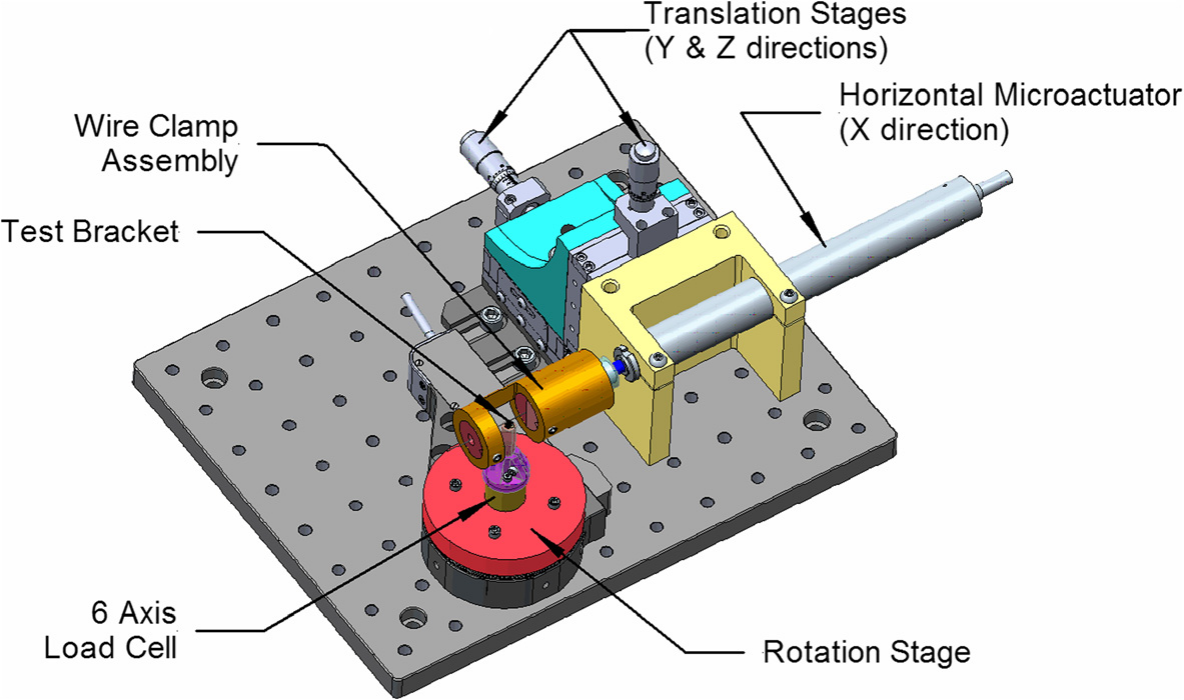
Orthodontic friction simulator (OFS) general view

**Fig. 3:**
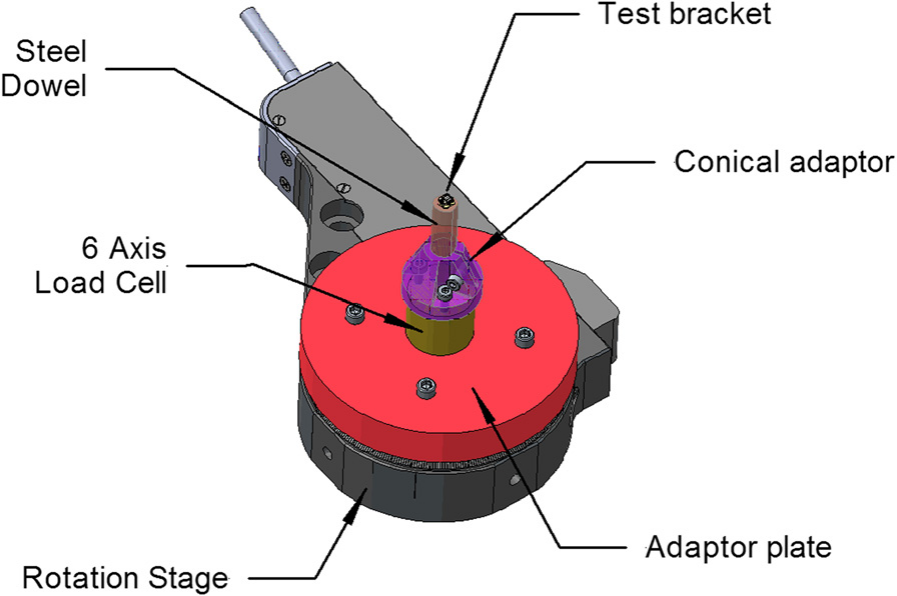
Detailed schematic of the bracket holder assembly and rotational stage

The orthodontic friction simulator (OFS) is shown in Figs. 2 and 3. Since forces involved are low, design goals were high precision, stiffness of the system, and portability.

The bracket is mounted on a stainless steel dowel inserted into an adaptor mounted on a six-axis load cell (Nano17® force transducer, ATI Industrial Automation, Apex, NC, USA). The dowel can be beveled to accommodate the torque prescription of the bracket, resulting in alignment of the bracket CSYS with the load cell. The sliding movement of an archwire through the bracket is achieved using a programmable motorized micrometer (microactuator) (M230.10 DC-Mike Actuator, Physik Instrumente (PI) GmbH & Co., Karlsruhe, Germany) and motor controller (C-863.10 Mercury TM DC motor controller, Physik Instrumente (PI) GmbH & Co., Karlsruhe, Germany) connected to a computer via a universal serial bus (USB) cable. The tip orientation of the bracket relative to the wire is obtained using a programmable rotating stage (PRM1Z8E, Rotation Mount with motor driver, Thorlabs, Newton, NJ, USA). Two manual translating stages (PT1, Translation Stage, Thorlabs, Newton, NJ, USA) are utilized to control the Y and Z position of the wire in the slot and hence either the wire offset or direct normal force applied by the wire on the bracket. The wire movement and bracket angulation are controlled digitally (acceleration, maximum velocity, and incremental move).

There are three significant differences between the device presented here and the one previously developed by Kamelchuk and Rossouw [30]. In the current apparatus, all six components of force and moment acting on the bracket are measured instead of only the pull force, *F*_*x*_. Previously, the wire was free on either side of the test bracket so that interbracket distance was not included [30]; this is incorporated in the present work. Finally, in previous work [30], the wire could be held under tension during the test and the amount of wire that could be pulled through the bracket was not limited. This is not implemented in the present apparatus, nor was it deemed necessary, as the wire tension in the oral environment is unknown.

**Fig. 4:**
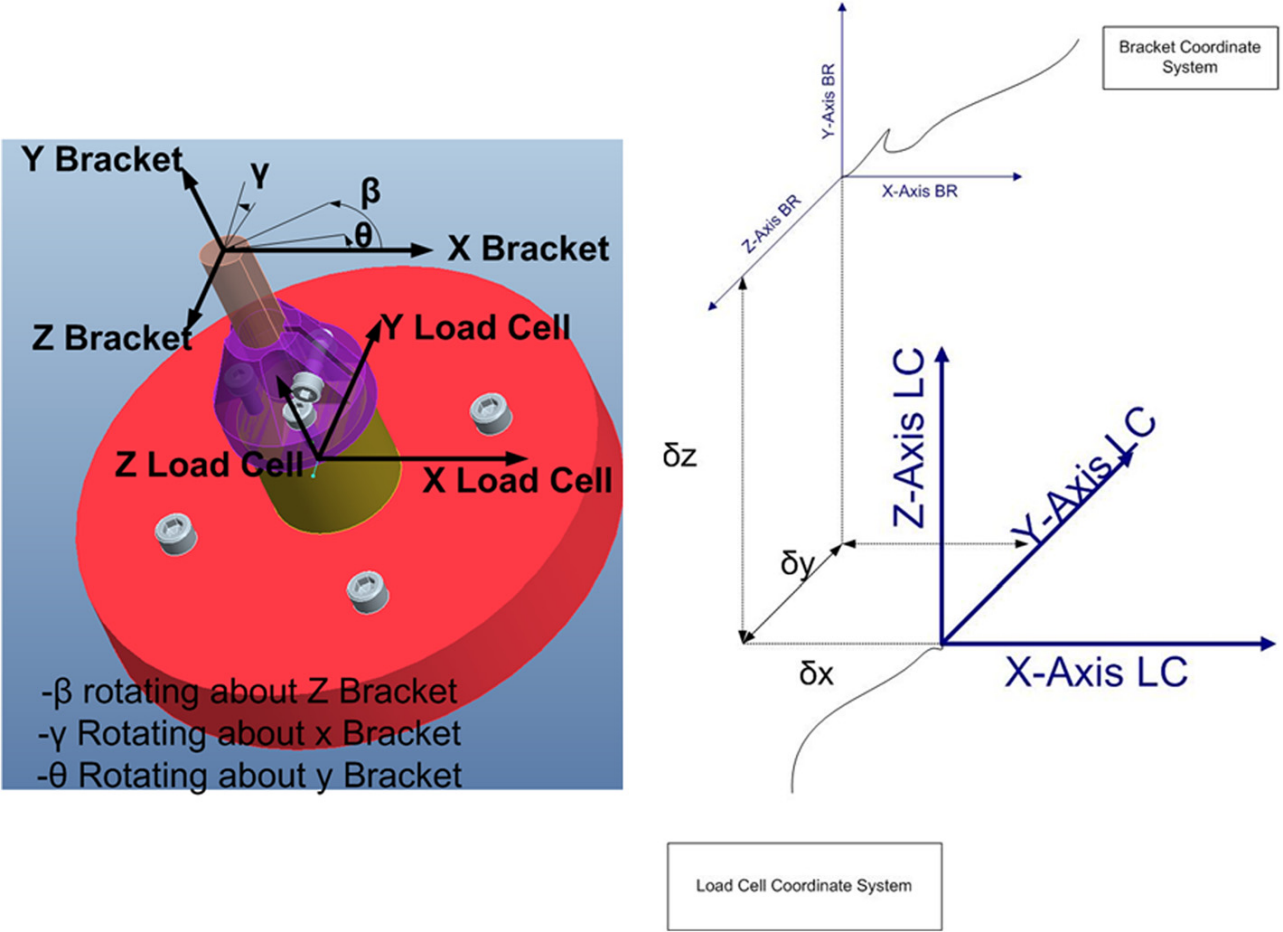
Representation of the coordinate system transformation from load cell to the bracket

A customized configuration file for each bracket contained settings determined by the testing protocol. These include motion settings for the actuators (linear and rotary) and for the data acquisition (NI-DAQ 6215, National Instruments, TX, USA) system (sampling speed and averaging settings, etc.). A custom computer program (LabWindows/ CVI, National Instruments, TX, USA) was written with a graphical user interface allowing for experiment automation and for real-time display of the experiment. Data is transformed from the load cell coordinate system to the bracket coordinate system and saved for later processing (Fig. 4).

### Sample preparation

Brackets were mounted on stainless steel cylindrical (1/4″ diameter, 1–1/4″ length) dowel pins (McMaster-Carr) using a porcelain conditioner (Reliance), primer (OrthoSolo, Universal Bond Enhancer), and composite resin application (Transbond XT, 3M Unitek). The top of the dowel was beveled at the manufacturer specified angle to compensate for any third-order torque prescription in the bracket; however, there was no compensation for other angular prescriptions, namely the first-order bracket angle of 5° for the Damon Q brackets. The rotation prescription for these brackets is 0°, and thus compensation is not required for this angle. Each dowel was machined with a locating flat surface in order to repeatedly maintain the orientation of the bracket and dowel relative to the load cell measurement coordinate system.

**Fig. 5:**
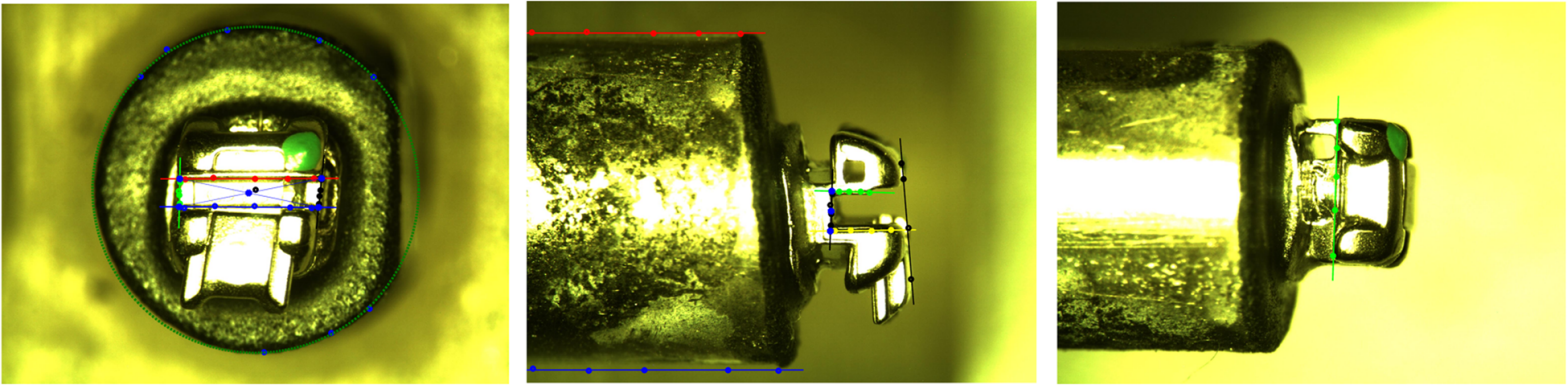
Top (left), side (middle), and front (right) views of a test bracket showing offset measurements

A CCD camera (Bausch & Lomb) captured three orthogonal views (Fig. 5) of each bracket/dowel pair. The image scales were approximately 0.005 mm/pixel. Custom software was used to determine the offset of the bracket CSYS relative to the coordinate system of the load cell at the base of the dowel. The (*x, y, z*) offset values and (θ*,* γ*,* β) bracket orientation angles for each bracket/dowel pair are stored in each bracket’s configuration file. This data was used to calculate the correct transformation of force/moment data from the load cell to the bracket coordinate system.

To prevent contamination of surfaces, ethanol was used to clean each bracket and archwire, and nitrile gloves were used throughout testing procedure. A new archwire was used for each bracket to avoid the introduction of wire surface damage or distortions caused by testing. For tests using elastic ligatures, a Straight Shooter Ligature Gun (TP Orthodontics) was used to standardize the force and stretching as much as possible. Because a new elastic was used for each bracket, effects of elastic stretch and aging were minimized.

Once the dowel was mounted in the load cell adaptor, a dissection microscope was used to precisely align the archwire in the center of the bracket slot using the manual translation stages. In addition to visualization of the archwire relative to the slot, the load cell data can be viewed in real time to ensure loads are negligible in the passive state. This adjustment also allows presetting the interaction force between the archwire and bracket if desired. The bracket was then ligated. For wet tests, 50 μL of human saliva was deposited on the bracket before and after wire ligation using a micro pipette for a total of 100 μL.

Wire speeds up to 1.2 mm/s are possible with the OFS, however, testing was conducted at 0.1 mm/s. All tests occurred over a distance of 1.1 mm. Bracket tip angle θ was incremented by 0.5° from 0° up to 5°. At each angle, the wire was moved 0.1 mm, travel time 1 s, during which force and moment data was collected at a sampling rate of 2000 Hz (50 samples/channel and with three channels each for forces and moments). This data was then averaged over every 50 samples and recorded in a log file for later processing.

The Damon Q (Ormco) left maxillary canine bracket with 0.018″ × 0.025″ stainless steel wires (Ormco) were used for this study. Damon Q brackets were used for both passive and elastomer ligation so that slot geometry remained consistent between trials. A sample size of 65 for elastic and self-ligating brackets in both the wet and dry state, yielding a total sample of 130, was used in this study. A new bracket, ligature (if required), and wire segment were used for each trial to remove any possible effects of wear and/or permanent deformation. Size 0.120 Power O Module (Ormco) elastic ligatures were used in this study. Brackets were randomly assigned to either be passively or elastically ligated prior to any testing, and the order of experiments was also randomized to remove bias.

### Error analysis

The potential system errors of the testing apparatus were quantified. With a 16-bit DAQ system, the resolution for the load cell was 0.003 N for force and 0.008 Nmm for moment. Errors could also arise in the transformation from the measured values at the load cell to the computed bracket values. With worst case offset errors, the overall transformation error was less than 0.8 % for *F*_*x*_, *F*_*z*_, and *M*_*y*_ and between 1.9–2.8 % for *F*_*y*_, *M*_*x*_, and *M*_*z*_. These marginal errors were acceptable.

Although manufacturing variations of brackets and wires exist, they are generally unknown to the clinician. Using Damon Q self-ligating bracket (slot = 0.022″, width = 0.110″) and 0.018″ × 0.025″ stainless steel archwire, the approximate theoretical θ_*c*_ is 2.08°. Manufacturing tolerance errors of 0.0005″ for archwire width, 0.0005″ for slot width, and 0.002″ for bracket width would produce θ_*c*_ in the range from 1.54° to 2.65°, contributing significantly to the variability in results. The variations in measured data are therefore more likely to arise from variations in bracket and wire geometry and elastic ligation force than from system errors in the friction apparatus itself.

## Results

**Fig. 6:**
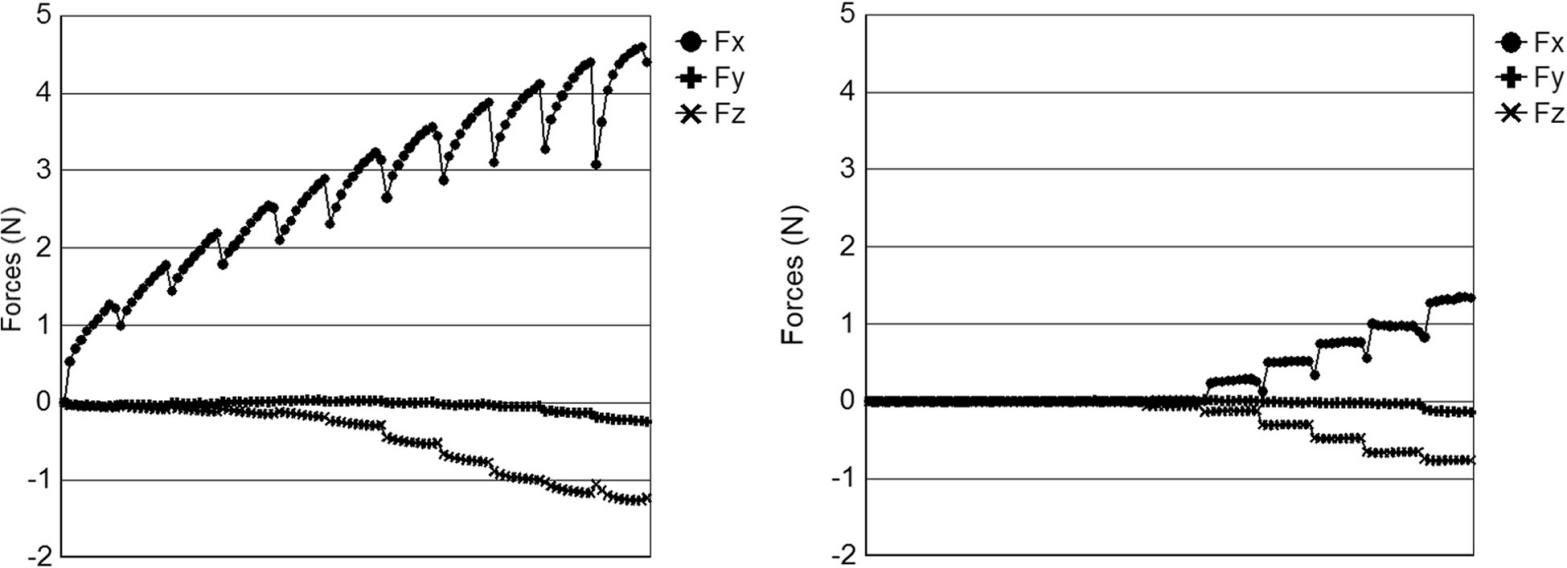
Typical force (N) versus wire travel (mm) plots from the OFS for elastic (left) and self-ligation (right) methods

**Fig. 7:**
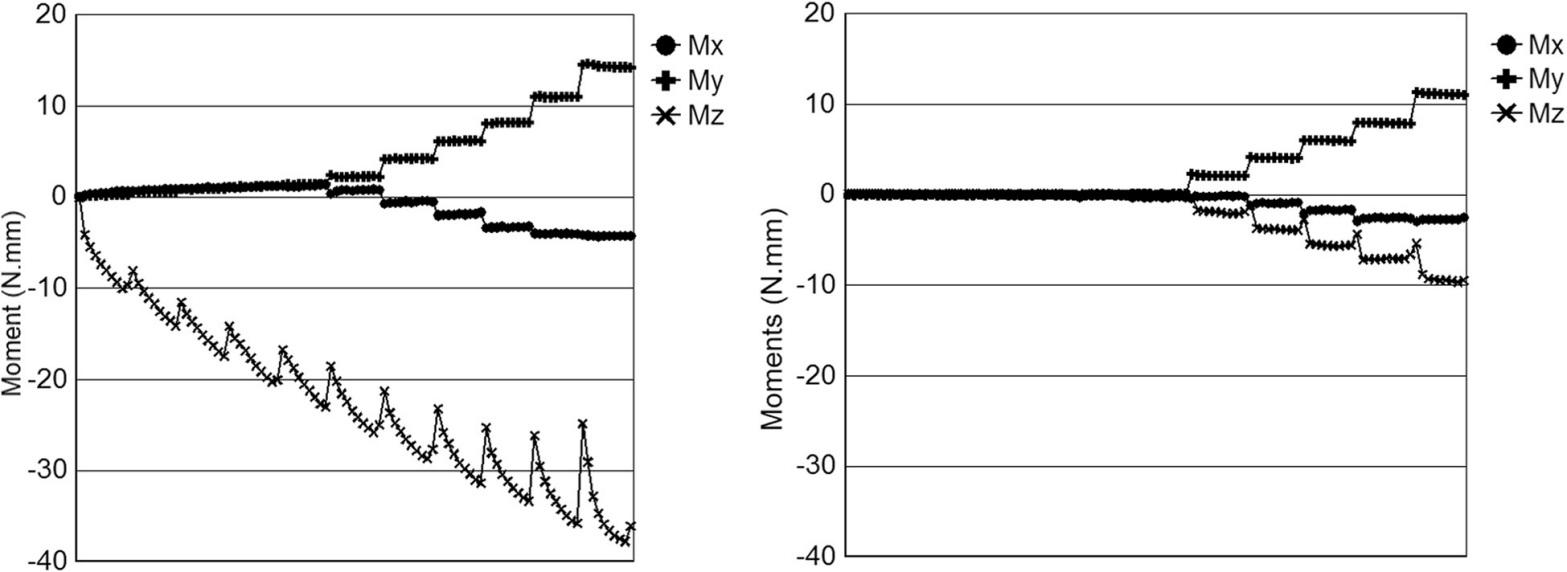
Typical moment (Nmm) versus wire travel (mm) plots from the OFS for elastic (left) and self-ligation (right) methods

Typical raw data sets for force, Fig. 6, and moment, Fig. 7, are presented considering only the dry state. The form of this data is typical of all tests. The horizontal axis represents the linear movement of the wire, and the stepped nature of the graphs corresponds to the incrementally changing tip angle. Of particular note in Fig. 6, however, is that for elastic ligation, the current test utilizing short incremental translations of the wire does not allow the forces (especially *F*_*x*_) to be fully expressed. Observing the test through a microscope, the stretching of the elastic during the test and a mild relapse at the end of each motion increment were noted. This means that the elastic ligation force is not constant through the test and has not reached its maximum. The measured force and moment components are therefore an underestimate of what might be considered a steady state value. This may be an advantage here since long continuous motion of the archwire does not occur clinically, and the ability of the elastic to “rebound” between incremental moves is probably closer to clinical reality.

**Fig. 8:**
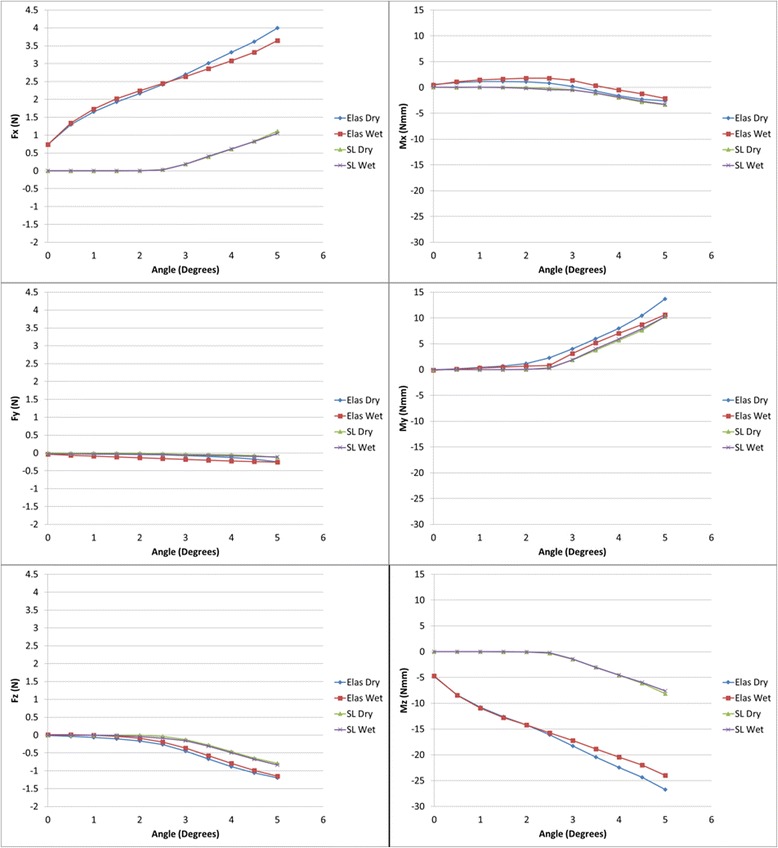
Average force components (left, top = X, middle = Y, bottom = Z) and moment components (right, top = X, middle = Y, bottom = Z) for all tests

Figure 8 shows the averaged data for all tests (*N* = 65 for both elastic and self-ligation). The general shape of these curves is the same as for the individual test data shown in Figs. 6 and 7, without the “stretching elastic” effect. It can be noticed that the magnitude of *M*_*z*_ increased throughout the experiment. This result is suspected to be due to the lack of bracket prescription compensation on the dowel in the first-order rotation direction. The minor change in *M*_*x*_ is also likely due to this factor as well.

**Fig. 9:**
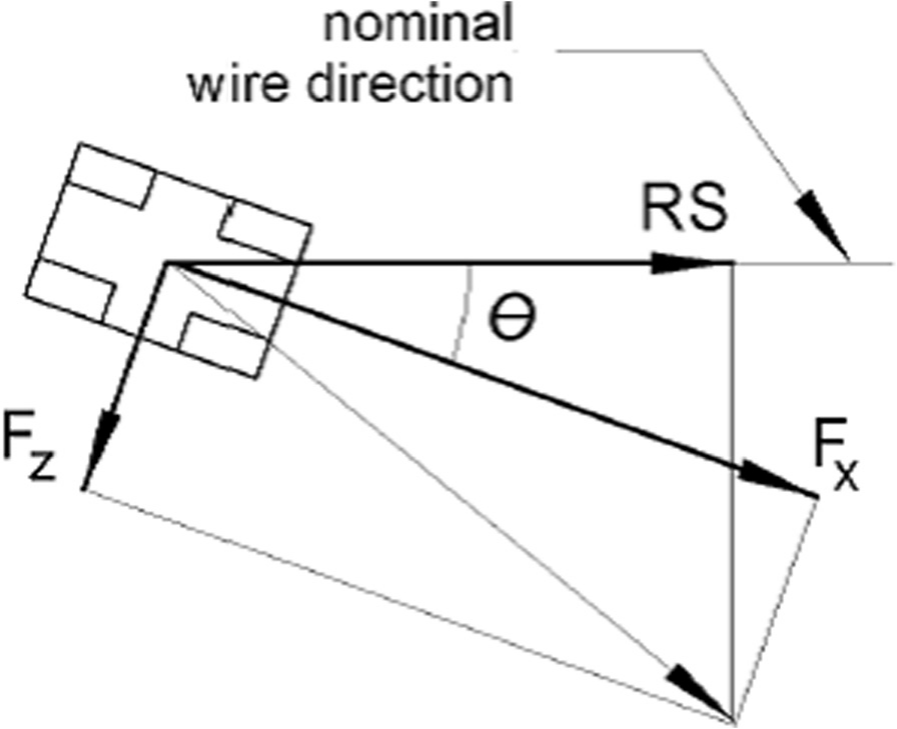
Definition of RS with positive values of Fx and Fz assumed

The wire pull direction is initially aligned with the bracket slot. As the bracket rotates, the wire pull direction is no longer purely in the X direction and a force component in the Z direction of the bracket will be generated (Fig. 9). Therefore, since RS is the total force in the nominal direction of the wire, it is calculated as Eq. (2):

2 $$ \mathrm{R}\mathrm{S}=\mathrm{F}\mathrm{x}*\  \cos\ \uptheta - \mathrm{F}\mathrm{z}\ * \sin\ \uptheta $$

**Fig. 10:**
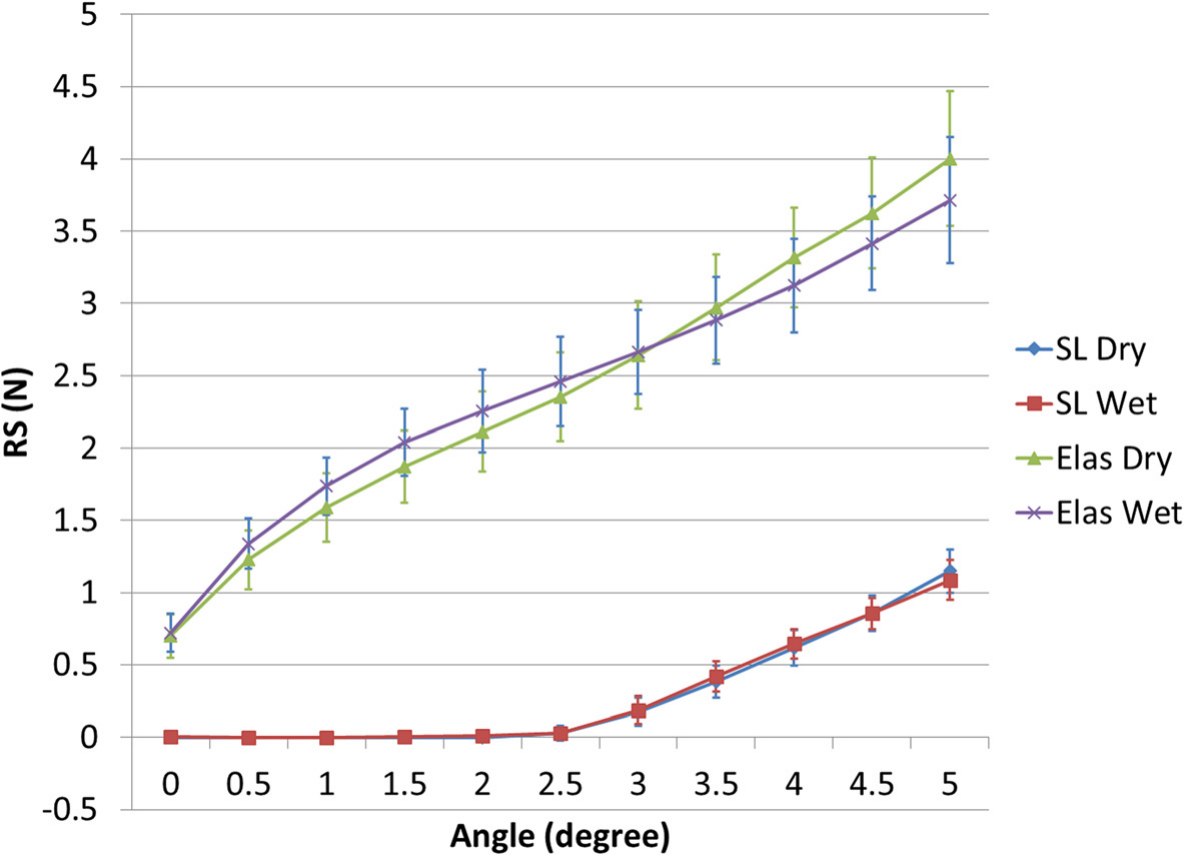
Average resistance to sliding (RS) and standard deviation for all test conditions

Note that the *F*_*z*_ components in Figs. 6, 7, and 8 are negative and so will actually increase *RS*. Although *F*_*y*_ is present, it is more-or-less perpendicular to the XZ plane and of very small magnitude; therefore, its contribution to *RS* is ignored. The combined average and standard deviation values of *RS* for all tests are shown in Fig. 10. The standard deviation of data tended to increase with angle. Note that the variation of the elastic ligation data is substantially greater than for self-ligation, undoubtedly due to variations in the ligation force of the elastics.

**Fig. 11:**
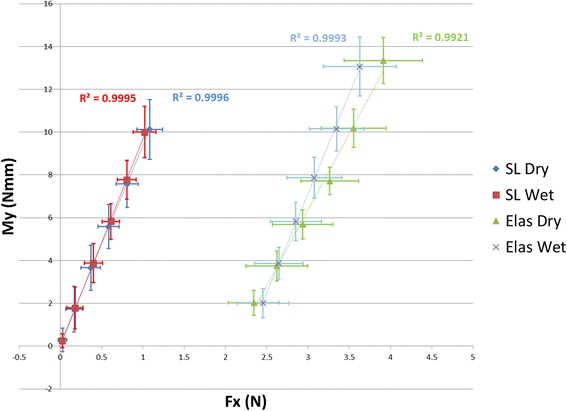
Relationship between My and Fx for binding, their correlation coefficients (R2), and respective standard deviations

Using data only for angulation above the critical contact angle for binding (2°), Fig. 11 illustrates *M*_*y*_ to be a linear function of *F*_*x*_ for both ligation methods. Since *M*_*y*_ = N*w, and using *F*_*x*_ = 2*μ_k_*N (because there are two contact points), the relationship between *M*_*y*_ and *F*_*x*_ can be expressed as Eq. (3):

3 $$ \mathrm{My}=\left(\mathrm{w}/2*\upmu \mathrm{k}\right)*\mathrm{F}\mathrm{x} $$

From linear regression of Fig. 11, μ_*k*_ values were determined and reported in Table 1.

**Table 1:** Coefficient of friction determined from binding data

	Coefficient of kinetic friction	
	Self-ligation	Elastic ligation
Dry	0.15	0.21
Wet	0.14	0.16

A repeated measures ANOVA was conducted (Table 2) to study the effect of state and ligation method on *F*_*x*_ and *M*_*y*_ values for angulations of 0° and 5°. When conducting experiments, five bracket de-bonded from the dowels and two additional brackets were omitted due to severe data corruption; thus, a total of 123 samples remained for statistical analysis. From the analysis, ligation method had a significant effect (*P* < 0.05) on *F*_*x*_ and *M*_*y*_ for both angles. Interestingly, the wet/dry state was found to have little effect on either value at 0° but made a significant contribution at 5°.

**Table 2:** Repeated measures ANOVA for each outcome and mean differences from pairwise comparisons (*n* = 123)

Outcome	Effects	F	*P*	η_p_^2^	Mean difference^a^
Fx 0°	State	0.001	0.982	4.0 × 10^−6^	NS
	State + elastomer	0.033	0.856	2.7 × 10^−4^	NS
	Elastomer	2904.2	<0.001*	0.96	0.74 N
Fx 5°	State	23.6	<0.001*	0.16	0.21 N
	State + elastomer	11.3	0.001*	0.09	0.30 N
	Elastomer	4281.9	<0.001*	0.97	2.74 N
My 0°	State	2.3	0.134	0.02	NS
	State + elastomer	0.2	0.685	0.001	NS
	Elastomer	4.0	0.047*	0.03	−0.05 Nmm
My 5°	State	3.9	0.050*	0.03	0.33 Nmm
	State + elastomer	3.7	0.056	0.03	NS
	Elastomer	282.3	<0.001*	0.70	3.12 Nmm

Degrees of freedom (1, 121) for each effect

*F* F statistic, *η*_*p*_^*2*^ partial eta squared, *NS* not significant

*P ≤ 0.05 was significant

^a^If *P* is significant, mean differences recorded from pairwise comparisons for state (dry–wet), elastomer (yes–no), and interaction of state + elastomer

## Discussion

### OFS performance

From the results presented in this manuscript, it is immediately apparent that the OFS improves upon existing experimental methods. Namely, this device is able to measure all the forces and moments acting at the bracket. At best, previous work was able to measure two components of force during friction tests. Through this capability, a relationship was derived relating *F*_*x*_ and *M*_*y*_ through the coefficient of kinetic friction, μ_*k*_. Previously, this would not have been possible as *M*_*y*_ at the bracket was not measureable.

Through the developed and programmable user interface, operators are able to easily and accurately control many test variables such as velocity, acceleration, and bracket rotation (in this case second-order rotation). This allows for a variety of experimental procedures to be set up quickly and performed repeatedly with minimal training. Given the amount of variables that may be manipulated with this apparatus, it was imperative that the controls remained as simple and straight forward as possible.

Finally, the OFS can be readily adapted to accommodate a variety of future hypotheses and experiments. For instance, by slightly offsetting the dowel and mounting the bracket on the side instead of on top, the effect of first-order rotation may be explored. In addition, by machining the tops of the dowels at various angles using the existing bracket position, the influence of third-order torque can be considered. Indeed, a number of future studies can be considered with only minimal adjustments to the OFS presented here.

### Effect of ligation method and dry/wet state

Based on the retrieved data, it is apparent that for self-ligation, since the wire is initially aligned with the slot, force and moment components are essentially zero until the onset of binding at approximately 2°. Once binding has occurred in self-ligating brackets, the force and moment values increased in a linear fashion with angle. This is in contrast to *F*_*y*_ and *M*_*x*_, however, which showed little variation with angulation, ligation method, or dry/wet state.

When considering elastic ligation, *FR* was not constant as expected due to the incremental stretching and rebound of the elastics. That is, the elastic tests never really reached a steady state. It is not clear, in fact, if that state would actually occur in vivo. Additionally, variations in measured forces and moments were considerably higher with elastic ligation than with self-ligation, undoubtedly due to inconsistency and variability in the elastomers or application technique.

While only *RS* has been analyzed by previous researchers, the role of binding illustrated by *M*_*y*_ can now be explored. It is evident that binding affects *M*_*y*_ for both self-ligated and elastically ligated brackets in a similar way: onset occurs at the binding angle and the rate of increase is similar for both types. Thus, the “uprighting moment” that is opposing the continued tip of the tooth is the same for both ligation methods. This has not been previously shown.

The differences due to dry versus wet testing were not significant in general based on the data. Using Fig. 11, there is negligible difference in μ_*k*_ values between dry and wet conditions for self-ligating brackets. Based on the results in Table 2, the state of the experiment had no effect on *F*_*x*_ or *M*_*y*_ at 0° angulation but did have a significant effect at 5°. While this would imply that the introduction of saliva could impact sliding mechanics at some point, in general, it was found to have negligible effect. The coefficient of kinetic friction for self-ligating bracket in this study was 0.15 (dry) and 0.14 (wet), which is comparable to 0.14 (dry) reported by Thorstenson and Kusy [10].

It is well known that interbracket distance has an effect on *RS* due to a change in effective stiffness of the wire [32]. The present system used an interbracket distance of approximately 6 mm, defined as the distance from the edge of the bracket to the edge of the clamp. This value varied slightly during the test due to the clamping system for the wire. In the current setup, the varying interbracket distance and wire misalignment could have contributed to the higher than expected observed *F*_*z*_ values. The ability to set a fixed desired interbracket distance as a test parameter is recommended for the next-generation 3D OFS. A more secure wire clamp design would ensure no rotational or lateral wire movements contributing to moment effects. A modified load cell adaptor would produce a smaller offset from the load cell to the bracket, which would allow a broader range of test conditions to be explored without overloading the load cell.

### Clinical significance

The presented study serves to explore fundamental relationships that exist both in the lab and in vivo. It is questionable whether informative lab experiments could be devised that involves all 20+ factors listed by Nanda [4]. This data is not available through any form of in vivo testing, nor would such testing be advisable since exploring the full range of variables would undoubtedly interfere with clinical treatment. It is desirable to maintain as many conditions fixed while varying only a few parameters in order to expose the correlation between those parameters. This sort of control is not available in vivo.

Laboratory findings, therefore, are meant to provide the underlying fundamentals that may then be considered in the expected clinical context. Only four of the many variables contributing to *RS* are explored with this system. These data, however, show the direct link between two of the force/moment components and the second-order tip. The critical contact angle is confirmed, as is a method for determining the effective coefficient of friction between the wire and the bracket.

Of particular note is the result that the couple *M*_*y*_ resulting from binding is the same for both elastic and self-ligation. This is the important uprighting moment that occurs during retraction in sliding mechanics.

This system measures kinetic friction on an isolated bracket in a steady state environment. Though clinical conditions are complicated to simulate, future studies evaluating both static and kinetic friction on a bracket experiencing oral functions, such as vibrations or vertical displacements, would be insightful. Similarly, since the compliance of the tooth mounted in the PDL will interact with the stiffness of the wire and hence the forces applied to the bracket, a future enhancement of the apparatus should include a method to simulate the PDL. An adjustable and constant interbracket distance would also be a desirable enhancement.

## Conclusions

The results reported here support Kusy and Whitley’s model for *RS* as composed of *FR* and *BI* components. The experimental θ_*c*_ value agreed with the theoretical equation based on bracket and wire geometry. Similar to Kusy and Whitley’s work for *F*_*x*_, above θ_*c*_, *RS* increased linearly with angulation for both self-ligation and elastic ligation. There was minimal effect of saliva in changing the frictional behavior of self-ligating bracket, while a small reduction in friction was observed under wet conditions with elastic ligation. Examination of all force and moment components acting on the bracket leads to additional insights into this complex interaction which was not previously possible. Current testing did not extend into the notching (*NO*) region due to issues with overloading the load cell.

The novelty of this 3D friction device includes measuring forces and moments in the X, Y, and Z directions applied on the bracket. These unique results, in addition to the marginal data errors, strengthen the utilization of this 3D device. Indeed, 3D simulations should be the standard protocol for understanding the friction phenomenon, as current 1D methods are limited in scope of information and application. The methodological variability between research teams makes comparisons between studies difficult or nearly impossible. As the interest in friction for orthodontic tooth movement continues, a goal of the research community should entail more standardized testing methods. This may include collaboration between research groups to use the same device and/or protocols.

## Abbreviations

RS: resistance to sliding
F: friction force
N: normal force
μ: general coefficient of friction
μs: static coefficient of friction
μk: kinetic coefficient of friction
CSYS: coordinate system located at the bracket
FR: simple, or classical, friction
BI: binding
NO: notching
Θ: second-order angle of the bracket
θ_c_: critical angle where the wire contacts bracket edges
OFS: orthodontic friction simulator
